# Dr. Dewitt Clinton Crumb

**Published:** 1908-06

**Authors:** 


Dr. Dewitt Clinton Crumb, of South Otselic, N. Y., died at his home April 18, 1908, aged 60 years. He graduated at the

University of Buffalo, medical department, in 1871; was a member of the medical societies of the State of New York and County of Chenango, and a veteran of the Civil War.

